# Nomogram to predict postpartum hemorrhage in cesarean delivery for twin pregnancies: a retrospective cohort study in China

**DOI:** 10.3389/fmed.2023.1139430

**Published:** 2023-04-18

**Authors:** Yanhua Zhang, Lu Chen, Weixiao Zhou, Jun Lin, Hong Wen

**Affiliations:** Department of Gynecological Oncology, Women's Hospital, School of Medicine, Zhejiang University, Hangzhou, Zhejiang, China

**Keywords:** postpartum hemorrhage, nomogram, cesarean delivery, twin pregnancies, prediction model

## Abstract

**Background:**

Postpartum hemorrhage (PPH) is the most common cause of maternal morbidity and mortality worldwide. A reliable risk assessment tool for PPH could optimize available interventions to reduce adverse maternal outcomes.

**Objective:**

The objective of this study was to explore a nomogram predicting the risk of postpartum hemorrhage after cesarean delivery for twin pregnancies.

**Methods:**

This single-center retrospective cohort study conducted twin pregnancies who underwent cesarean delivery between January 2014 and July 2021. Propensity score matching at baseline was used to match PPH (blood loss ≥1000 mL) and non-PPH group (blood loss <1000 mL). A nomogram was developed to predict the risk of PPH in cesarean delivery for twin pregnancies. The receiver operating characteristic curve (ROC), calibration plot, and decision curve analysis (DCA) were, respectively, used to evaluate the discrimination, calibration, and clinical utility of the prediction models.

**Results:**

After propensity score matching, 186 twin pregnancies in the PPH group were matched with 186 controls in the non-PPH group. Seven independent prognostic variables, including antepartum albumin, assisted reproductive technology, hypertensive disorders of pregnancy, placenta previa, placenta accrete spectrum, intrapartum cesarean delivered, and estimated weights of twins, were used to build the nomogram. Based on the performance of the model, it appears that a good calibration (Hosmer–Lemeshow χ^2^ = 4.84, *P* > 0.05), an excellent predictive ability (area under the curve: 0.778, 95% CI: 0.732–0.825), and a good positive net benefit in the predictive model have been achieved.

**Conclusion:**

The nomogram was first generated to predict PPH in cesarean delivery for twin pregnancies, which could help clinicians to provide a reference for the preoperative surgical plan, choose optimal treatments, optimize healthcare resources, and thereby reduce the associated adverse maternal outcomes.

## Introduction

Globally, postpartum hemorrhage is the leading cause of maternal mortality and morbidity, accounting for nearly one-third of all maternal deaths (1, 2). With the widespread availability of ART, the incidence of multiple pregnancies is increasing, accompanying with obstetric complications. Compared with singleton pregnancies, twin pregnancies have a higher risk of PPH. Hence, the perinatal management of twin pregnancies needs to receive high priority (3–7).

The purpose of this study was to develop a model that can predict the risk of postpartum hemorrhage after cesarean delivery in twin pregnancies using clinical data, identify women at risk of PPH, and improve the capability of PPH prediction. The model was incorporated into a specially designed nomogram that could be used as a tool to optimize the available interventions to reduce negative maternal outcomes.

## Methods

### Study population

**Figure 1:**
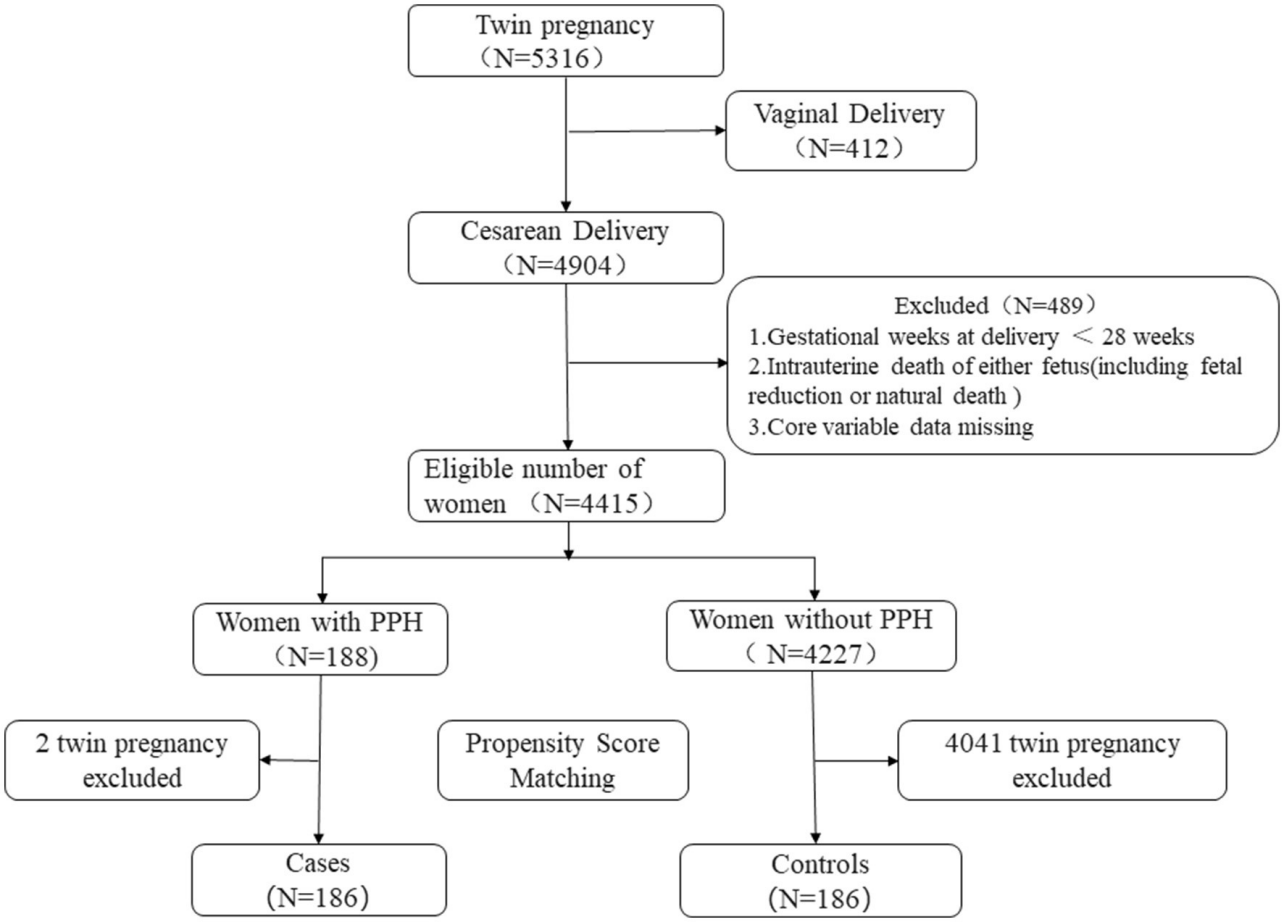
Study flow diagram.

Among the study population were all women who had cesarean deliveries at Women's Hospital School of Medicine Zhejiang University between January 2014 and July 2021. The exclusion criteria included vaginal delivery, gestational weeks at delivery < 28, core variable data missing, and intrauterine death of either fetus (including fetal reduction or natural death). Propensity score matching was used to match twins with PPH in a 1:1 ratio to the twins without PPH. The final dataset included 186 twin pregnancies with PPH and 186 propensity score-matched controls with non-PPH (Figure 1).

### Data collection

– Primary outcome variable: According to the classifications of the American College of Obstetricians and Gynecologists, PPH is defined as estimated blood loss (EBL) ≥ 1000 mL within 24 h after delivery, regardless of delivery mode (8). To improve accuracy, a weighing method was used for hemorrhage evaluation during the 24 h following delivery. During the operation, the amount of blood loss was quantified by measuring the blood in gauze and the fluid collected via the suction tube. All women giving birth should be offered conventional IM/IV oxytocin (10 IU) after the delivery of the placenta to prevent PPH. If persistent bleeding, the use of additional oxytocin (10 IU, IV/IM) and other injectable uterotonics (i.e., carbetocin or carboprost trometamol) was recommended.

– Independent variables were as follows:
• Maternal: maternal age, gravidity, parity, pre-pregnancy body mass index (BMI), gestational age at delivery, history of intrauterine surgery, assisted reproductive technology (ART), antepartum hemoglobin, and antepartum albumin.
• Obstetric: premature rupture of membranes (PROM), diabetes mellitus (pre-gestational or gestational) (DM), hypertensive disorders of pregnancy (HDP, including chronic hypertension, gestational hypertension, preeclampsia, or eclampsia), placental abruption, placenta previa, placenta accreta spectrum (PAS), and intrapartum cesarean delivery.
• Fetal: chorionicity of twins; estimate the fetal weight (EFW) of twins.

### Definitions for analysis

The continuous variables were categorized based on the clinical cutoff value. Parity was classified as primipara and multipara status. Maternal age is divided into two groups according to advanced age or not (<35 years or ≥ 35 years). Chorionicity of twins includes dichorionic-diamniotic (DCDA), monochorionic-diamniotic (MCDA), and monochorionic-monoamniotic (MCMA). Intrapartum cesarean delivery was referred to as a cesarean delivery performed after the onset of labor or induction of labor (9). Gestational age at delivery was divided into three groups: 28–33 + ^6^ weeks, 34–36 + ^6^ weeks, and >37 weeks. The EFW of twins was classified into three groups: <4000 g, 4000–4999g, and ≥5000g. Pre-pregnancy BMI was classified as follows: underweight (BMI <18.5 kg/m^2^), normal (18.5–23.99 kg/m^2^, reference), overweight (24–27.99 kg/m^2^), and obese (BMI ≥28 kg/m^2^) according to the WHO classification; (10). PAS introduced by FIGO in 2018 was defined abnormal adhesion and invasive placenta (11).

### Statistical analysis

Propensity scores were calculated using logistic regression with PPH as the dependent variable. Controls were selected using propensity-matching methods based on multiple confounders simultaneously. With a caliper width of 0.3 of the standard deviation of the propensity score, propensity score matching was performed using a 1:1 matching protocol without replacement (greedy-matching algorithm).

Continuous variables were expressed as mean ± SD or interquartile range (IQR) depending on their normality. Categorical variables were expressed as numbers (percentages). The analysis was carried out by using Student's *t*-test or the ANOVA for continuous variates and the chi-square test/Fisher's exact test for categorical variates, respectively. The variates with *p* ≤ 0.1 in the univariate logistic regression analysis and a factor that did not meet the standards in the univariate analysis but was clinically significant were selected for the multivariable analysis, in which forward stepwise algorithms were conducted. The performance of the multivariable logistic regression-based nomogram was evaluated by the discrimination and calibration, using the ROC curve and its area under the curve (AUC) to illustrate its discriminating power, while a calibration curve and the Hosmer–Lemeshow (H-L) test was used to determine the adequacy of calibration. A bootstrap sampling method (Bootstrap) was used to internally validate the model. With bootstrapping, the calibration plot illustrated the association between predicted and actual probabilities, which reflected the nomogram's performance characteristics. Decision curve analyses (DCA) were employed to demonstrate the clinical usefulness of the predictive nomogram model by estimating the net benefits over a spectrum of probability thresholds. Statistical significance was defined as *p* < 0.05 with two-sided analysis. All data management and statistical analyses were accomplished by IBM SPSS Statistics (version 20.0) and R software (version 3.6.1).

## Results

### Patient characteristics

There was no statistically significant difference between the two groups in maternal age, gravidity, parity, gestational age at delivery, pre-pregnancy BMI, DM, placental abruption, chorionicity, history of intrauterine surgery, and scarred uterus. The median amount of PPH was 1350 ml and 300 ml in both groups, respectively (Table 1).

**Table 1:** Baseline characteristics of propensity score-matched PPH study cohort with and non-PPH control group.

	**PPH group (*N =* 186)**	**non-PPH group (*N =* 186)**	***P-*** **value**
Maternal age (year)	31.01 ± 3.80	31.61 ± 3.88	0.131
Amount of PPH	1,350 (893)	300 (100)	<0.001
Gravidity			0.302
1	78 (41.9)	91 (48.9)	
2	58 (31.2)	46 (24.7)	
≥3	50 (26.9)	49 (26.3)	
Parity			0.805
Nulliparous	145 (78.0)	142 (76.3)	
Multipara	41 (22.0)	44 (23.7)	
Gestational age at delivery			0.392
28–33^+6^	42 (22.6)	33 (17.7)	
34–36^+6^	101 (54.3)	101 (54.3)	
≥37	43 (23.1)	52 (28.0)	
Pre-pregnancy BMI (kg/m^2^)			0.260
<18.5	18 (9.7)	26 (14.0)	
18.5–24.9	142 (76.3)	141 (75.8)	
25–29.9	25 (13.4)	16 (8.6)	
≥30	1 (0.5)	3 (1.6)	
History of intrauterine surgery			0.602
No	126 (61.2)	119 (64.0)	
Yes	80 (38.8)	67 (36.0)	
Scarred uterus			0.770
No	160 (86.0)	157 (84.4)	
Yes	26 (14.0)	29 (15.6)	

BMI, body mass index. Data are shown as numbers (%) or IQR.

### Analysis of risk factors for PPH between two groups

Univariate analysis showed that twin pregnancies with PPH were more likely to have HDP (24.7 vs. 14.0%), placenta previa (29.0 vs. 1.6%), PAS (21.5 vs. 3.2%), anemic (54.% vs. 71.5%), intrapartum CD (33.9 vs. 17.2%), and hypoproteinemia compared with those with non-PPH (Table 2).

**Table 2:** Univariate logistic regression analysis of the risk factors for PPH after cesarean delivery of twin pregnancies.

	**PPH group (*N =* 186)**	**non-PPH group (*N =* 186)**	***P-*** **value**
Antepartum hemoglobin (g/l)			<0.001
< 110	84 (45.2)	53 (28.5)	
≥110	102 (54.8)	133 (71.5)	
Antepartum albumin (g/l)	31.1(4.9)	32.1(3.7)	0.002
EFW of twins (g)			0.642
< 4000	43 (23.1)	41 (22.0)	
4000–4999	68 (36.6)	77 (41.4)	
≥5000	75 (40.3)	68 (36.6)	
Chorionicity			0.239
DCDA	164 (87.1)	149 (80.1)	
MCDA	22 (11.8)	36 (19.4)	
MCMA	2 (1.1)	1 (0.5)	
ART			0.068
No	60 (32.3)	78 (41.9)	
Yes	126 (67.7)	108 (58.1)	
HDP			0.012
No	140 (75.3)	160 (86.0)	
Yes	46 (24.7)	26 (14.0)	
Placenta previa			<0.001
No	132 (71.0)	183 (98.4)	
Yes	54 (29.0)	3 (1.6)	
PAS			<0.001
No	146 (78.5)	180 (96.8)	
Yes	40 (21.5)	6 (3.2)	
Intrapartum CD			<0.001
No	123 (66.1)	154 (82.8)	
Yes	63 (33.9)	32 (17.2)	
Diabetes mellitus			1.000
No	139 (74.7)	140(75.3)	
Yes	47 (25.3)	46 (24.7)	
Placental abruption			
No	180 (96.8)	184 (98.9)	0.284
Yes	6 (3.2)	2 (1.1)	
PROM			1.000
No	162 (87.1)	162 (87.1)	
Yes	24 (12.9)	25 (86.6)	

ART, assisted reproductive technology; CD, cesarean delivery; HDP, hypertensive disorders of pregnancy; PAS, placenta accreta spectrum; PROM, premature rupture of membranes; MCMA, monochorionic-monoamniotic; MCDA, monochorionic-diamniotic; DCDA, dichorionic-diamniotic. Data are shown as numbers (%) or IQR.

The subsequent multivariable analysis revealed that antepartum albumin [OR = 0.92, 95% CI (0.86–0.99)], ART [OR = 1.81, 95% CI (1.08–3.03)], HDP [OR = 2.03, 95% CI (1.06–3.91)], placenta previa [OR = 28.15, 95% CI (7.95–99.76)], PAS [OR = 4.97, 95% CI (1.85–13.32)], intrapartum CD [OR = 2.48, 95% CI (1.40–4.37)], and EFW of twins were significantly related to PPH in the overall cohort. The detailed results are shown in Table 3.

**Table 3:** Multivariable logistic regression analyses of the risk factors for PPH after cesarean delivery of twin pregnancies.

	**OR (95% CI)**	***P***
Antepartum hemoglobin (g/l)	1.63 (0.96–2.76)	0.071
Antepartum albumin (g/l)	0.92 (0.86–0.99)	0.037
EFW of twins (g)		0.002
<4000	Reference	
4000–4999	1.89 (0.91–3.91)	0.086
≥5000	3.47 (1.67–7.21)	<0.001
ART	1.81 (1.08–3.03)	0.024
HDP	2.03 (1.06–3.91)	0.033
Placenta previa	28.15 (7.95–99.76)	<0.001
PAS	4.97 (1.85–13.32)	0.001
Intrapartum CD	2.48 (1.40–4.37)	0.002

ART, assisted reproductive technology; CD, cesarean delivery; HDP, hypertensive disorders of pregnancy; PAS, placenta accreta spectrum; MCMA, monochorionic-monoamniotic; MCDA, monochorionic-diamniotic; DCDA, dichorionic-diamniotic.

### Development of an individualized prognostic model

**Figure 2:**
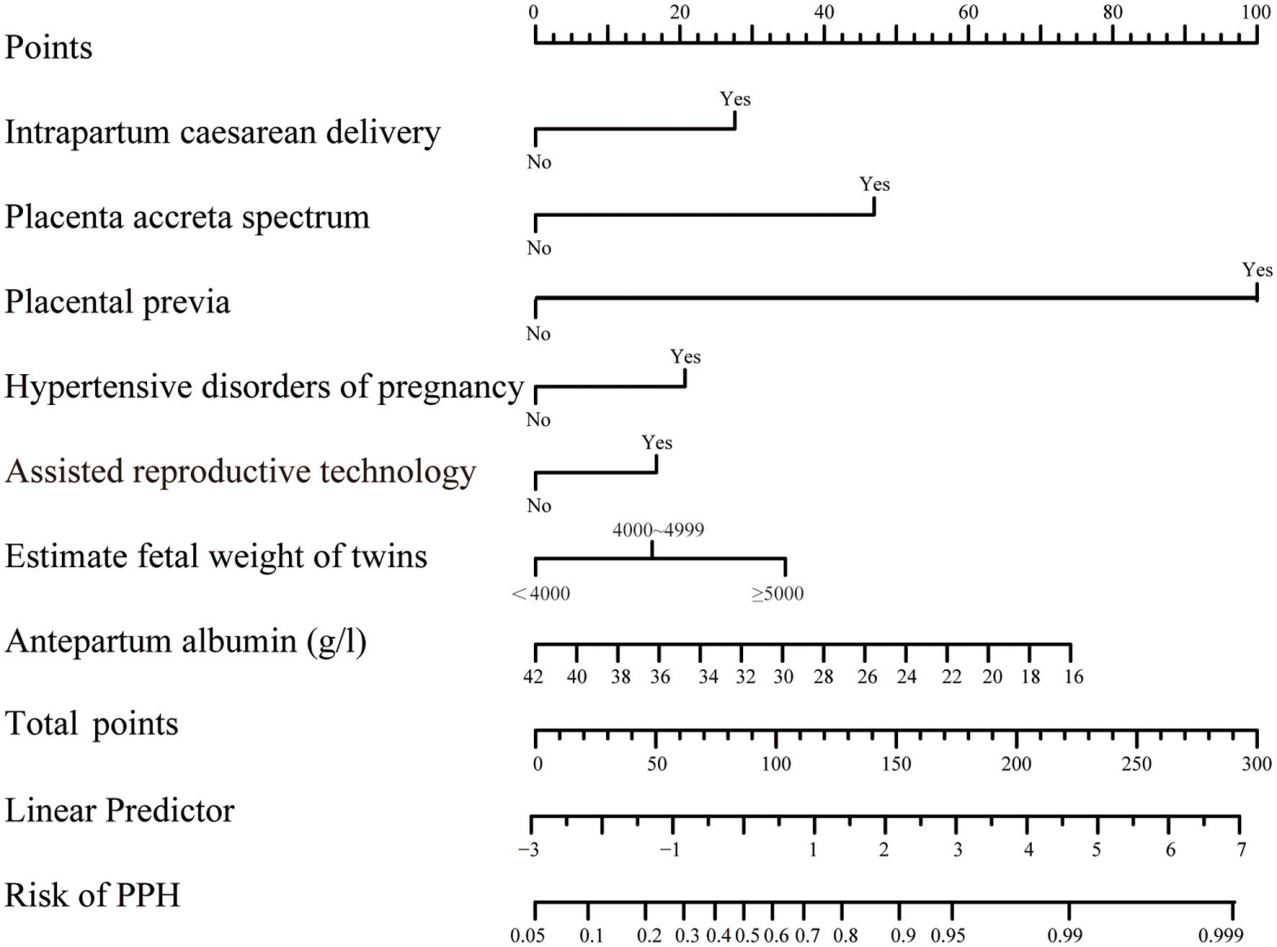
Nomograms for twin pregnancies in PPH probability estimate. The score of each variable was obtained by drawing a vertical line from the corresponding value to the top point axis separately, and then, the cumulative point score for all the variables was matched to the corresponding risk of postpartum hemorrhage.

A nomogram was developed to predict the risk of PPH based on the model generated by multi-factor logistic regression analysis (Figure 2). For each variable value, we can draw an upward vertical line along the point axis and then sum all those points, and then, we can draw a downward vertical line from the “Total Points” line to estimate the risk of PPH.

### Validation of the nomogram and its clinical usefulness

**Figure 3:**
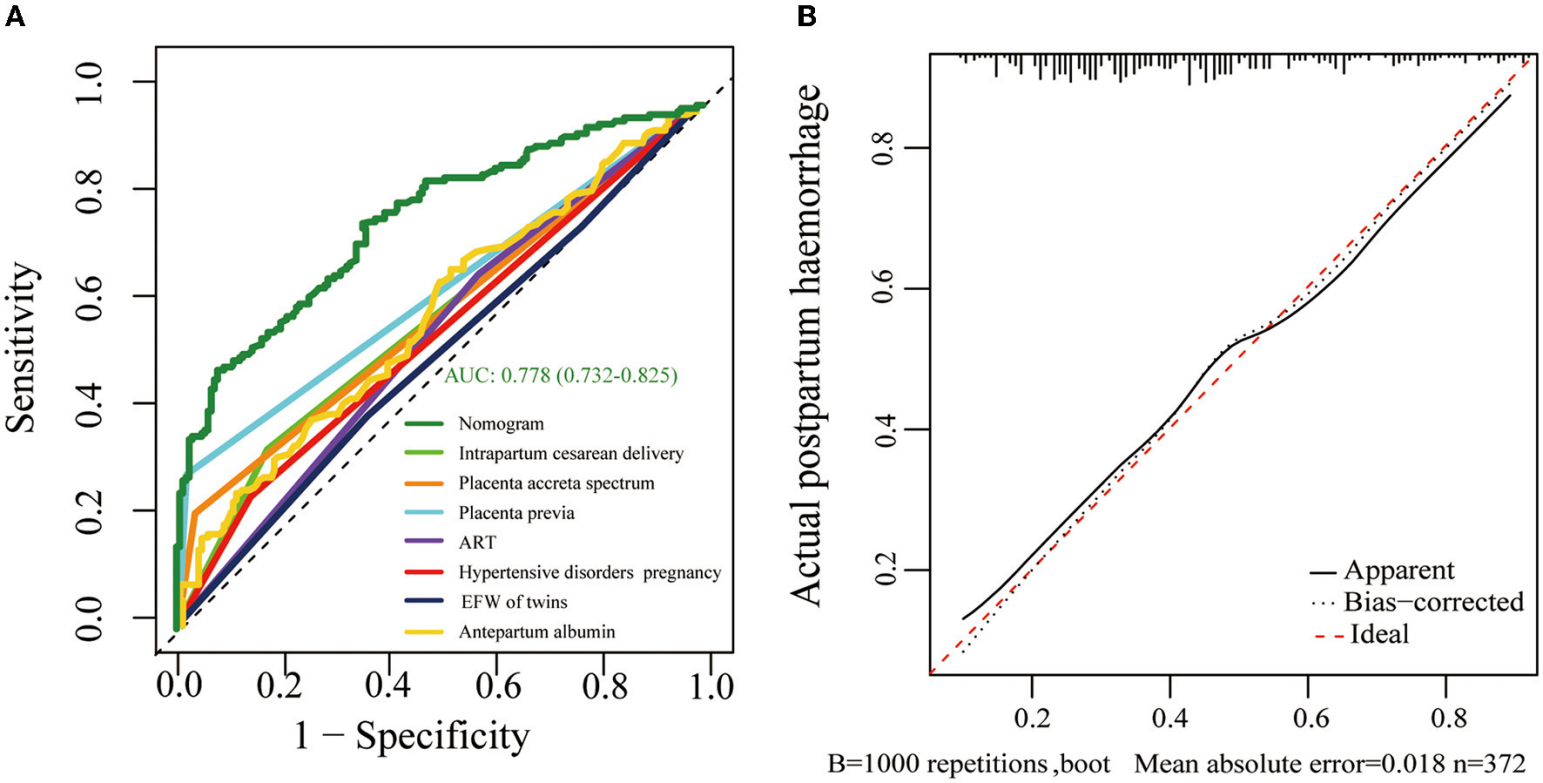
**(A)** Receiver operating characteristic (ROC) curves of the formulated nomogram model. The nomogram of the area under the receiver operating characteristic curve (AUC) was 0.778 (95% CI 0.732–0.825). **(B)** Calibration curve delineates the goodness of fit between the predicted probability and the actual probability. The horizontal axis represents the predicted risk of postpartum hemorrhage, and the vertical axis represents the observed outcome of the event. The diagonal dashed line signifies the ideal prediction, and the dotted line shows the predictive performance of the nomogram.

**Figure 4:**
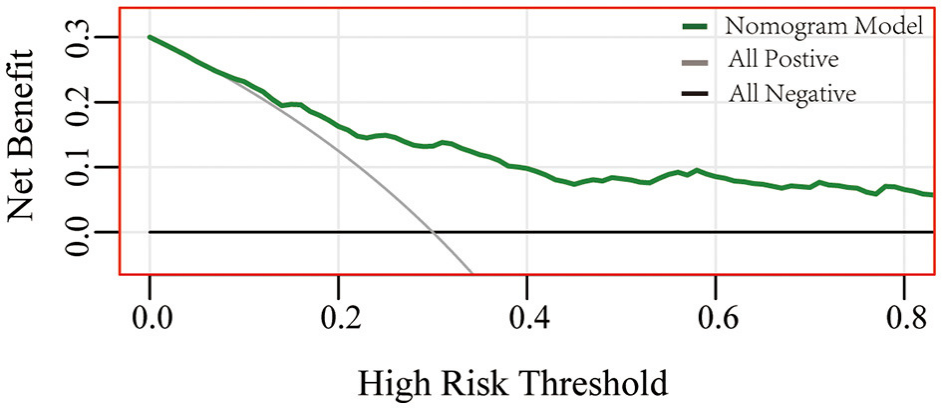
Performance of decision curve analysis. Decision curve analysis demonstrates the theoretical relationship between the net benefit of the model across different threshold probabilities (12). The horizontal axis represents the threshold risk of twin pregnancies patients who happened to have postpartum hemorrhage. The gray line assumes that all patients happened to postpartum hemorrhage, and the black thin line assumes no patients happened to postpartum hemorrhage. At the same threshold probability, the larger net benefit implies that more benefits patients can achieve using the model.

A nomogram based on forward stepwise multiple logistic regression was developed to estimate the probability of PPH during cesarean delivery for twin pregnancies (Figure 2). According to the ROC curve analysis, this nomogram performed well as a diagnostic model with an AUC value of 0.778, with the sensitivity and specificity of 68.3 and 78.5%, respectively (Figure 3A). The prediction effect of the model was verified using bootstrap self-sampling with 1000 replicates, and the result was visualized in the calibration curve, which demonstrated a good agreement between prediction and observation in the dataset (χ^2^ = 4.843, *p* > 0.05), implying that the nomograms predicting were well-calibrated (Figure 3B). According to our decision curve analysis, our predictive model showed good benefits for different threshold probabilities, which demonstrated a favorable clinical effect in the predictive model (Figure 4).

## Discussion

In the world, postpartum hemorrhage is the leading cause of maternal mortality, accounting for 19.9 to 36.2% of all maternal deaths (1). The risk of PPH is significantly higher in twin pregnancies than in singleton pregnancies. Studies have reported that the incidence of PPH in multiple gestations is about 2.26 to 4 times higher than that in singleton pregnancies (13, 14). The high incidence of PPH in twin pregnancies makes predicting the risk of PPH extremely important from a clinical perspective. Therefore, early detection and appropriate interference of PPH without delay are crucial in preventing maternal death. In this study, we successfully developed a risk prediction model for predicting PPH in cesarean delivery for twin pregnancies, which enables prediction of the incidence of PPH before delivery to facilitate assessment and guide individualized treatment.

According to previous reports, numerous prenatal risk factors are associated with PPH (15–22). In this study, factors that could potentially affect PPH in twin pregnancies were statistically analyzed, and regression analysis revealed that HDP, placenta previa, intrapartum cesarean delivery, ART, and PAS were significantly associated with postpartum hemorrhage in twin pregnancies, which is consistent with the results of previous studies on risk factors for PPH in singleton pregnancies (9, 23–26). In addition, the results of this study suggest that antepartum albumin and EFW of twins were risk factors for postpartum hemorrhage in twin pregnancies. Antepartum hypoproteinemia may cause myometrial edema, which may be associated with uterine atony, increasing the risk of PPH (27, 28). Estimated fetal weight of twins, which has important clinical implications for PPH, is related to overdistention of the uterus, leading, in turn, to impair myometrial contractility retractability of the uterine muscles after delivery, which increases the incidence of PPH.

Although there are many studies evaluating factors that influence PPH, no appropriate predictive models could be available for clinical application for twin pregnancies. Neary et al. conducted a systematic review of 14 published prediction models for PPH, and the results showed that three of them had some clinical application potential. However, these three models were limited by their applicable population who experienced CS, placenta accreta spectrum disorders with MRI placental evaluation, or placenta previa patients (29). Blitz MJ et al. developed a model to predict PPH for twin pregnancies, which listed PPH requiring the administration of packed red blood cells (PPH + PRBC) as an outcome. However, there were some subjective factors in the decision to administer a PRBC transfusion to some extent (5). Compared to these prior models, this study first established a nomogram to predict the risk of PPH in cesarean delivery for twin pregnancies. The results show a good calibration (Hosmer–Lemeshow χ^2^ = 4.84, *P* > 0.05), an excellent predictive ability (AUC: 0.778, 95% CI: 0.732–0.825), and a good positive net benefit.

Placenta previa and placenta accrete spectrum, which acts as the strongest predictor for PPH, may result in severe PPH or even a hysterectomy (30, 31). In recent years, intravascular balloon occlusion has become an increasingly popular prophylactic measure, which includes intravascular balloon occlusion of the abdominal aorta or internal iliac arteries (32–36). During the surgery, the abdominal aorta/internal iliac artery can be temporarily blocked by the balloon, thus reducing the uterine artery's blood supply, and the hemostatic procedures can be simplified and shortened. However, intravascular balloon occlusion may also cause complications, such as thrombosis diseases, hematoma, rare artery rupture, and high healthcare costs. Given these, only for patients with very high-risk PPH, such as pernicious placenta previa complicated with placenta accreta, the technique is performed in our hospital. There were three patients were diagnosed with pernicious placenta previa complicating with placenta accreta after intravascular balloon occlusion performed in our hospital in 2015. Two of them were terminated at 28 weeks of gestation without intravascular balloon occlusion because of the low benefit of the intravascular balloon occlusion technique given the small gestational weeks. In the other case, the emergency CD termination was chosen without the intravascular balloon occlusion technique at 35 weeks because of high vaginal bleeding after contractions accompanied by unstable vital signs. We hope to introduce related data about intravascular balloon occlusion in twin pregnancies in the future.

When a high-risk patient with PPH is identified, reasonable preventive measures can be taken, such as ameliorating of patient's antepartum albumin, timeous operative delivery in cases of prolonged labor, meticulous surgical technique, intraoperative cell salvage, and the preparation of additional plasma or serum in advance. Extremely high-risk patients, such as pernicious placenta previa or placenta accrete spectrum, may be considered for prophylactic arterial balloon occlusion to reduce the rate of blood loss. However, many of the seven influencing factors could not be modified, and what we can do is make an adequate preoperative preparation and work up by an experienced multidisciplinary team.

## Strengths and limitations of the study

By using nomograms, it is possible to implement complex logistic regression models in a convenient visual way, and nomograms are widely used in clinical events. As of now, predictive models for PPH have largely been developed for singleton pregnancies, and fewer have been developed for twin pregnancies. In our study, we first developed the prediction models for PPH in cesarean delivery for twin pregnancies, and the model can accurately quantify an individual's comprehensive risk of PPH with favorable discrimination, calibration, and positive net benefit.

Our study had some limitations. First, the main limitation in our study related directly to its retrospective nature, and limitation bias might be present. Second, this is a single-center study, and all subjects are limited to a single hospital, which does not reflect most women with twin pregnancies. Lastly, the study lacked external validation, more data on PPH should be included, and external validation should be performed to demonstrate the predictive ability of the nomogram in further studies.

Our study is the first to construct a nomogram for predicting postpartum hemorrhage in cesarean delivery for twin pregnancies, which exhibited a predictive performance. The nomogram can be applied in clinical practice, if the result of external verification is satisfactory, which may help accurately predict the risk of PPH and provide a reference for targeted intervention measures, thereby reducing the associated adverse maternal outcomes.

## Data availability statement

The raw data supporting the conclusions of this article will be made available by the authors, without undue reservation.

## Ethics statement

The studies involving human participants were reviewed and approved by the Women's Hospital School of Medicine Zhejiang University. Written informed consent for participation was not required for this study in accordance with the national legislation and the institutional requirements.

## Author contributions

YZ was responsible for study conceptualization and design, data curation and analysis, and writing the manuscript. WZ and LC were responsible for acquiring data, interpreting it, and determining the methodology. JL helped with the design of the study and critical revision of the manuscript. HW contributed to the design of the study, gathering, analyzing data, reviewing, and editing the manuscript. All authors have read and approved the published version of the manuscript.
